# Optimization of On-Demand Shared Autonomous Vehicle Deployments Utilizing Reinforcement Learning

**DOI:** 10.3390/s22218317

**Published:** 2022-10-29

**Authors:** Karina Meneses-Cime, Bilin Aksun Guvenc, Levent Guvenc

**Affiliations:** Automated Driving Lab, Ohio State University, Columbus, OH 43210, USA

**Keywords:** shared autonomous vehicles, mobility, traffic-in-the-loop simulation, optimization, reinforcement learning

## Abstract

Ride-hailed shared autonomous vehicles (SAV) have emerged recently as an economically feasible way of introducing autonomous driving technologies while serving the mobility needs of under-served communities. There has also been corresponding research work on optimization of the operation of these SAVs. However, the current state-of-the-art research in this area treats very simple networks, neglecting the effect of a realistic other traffic representation, and is not useful for planning deployments of SAV service. In contrast, this paper utilizes a recent autonomous shuttle deployment site in Columbus, Ohio, as a basis for mobility studies and the optimization of SAV fleet deployment. Furthermore, this paper creates an SAV dispatcher based on reinforcement learning (RL) to minimize passenger wait time and to maximize the number of passengers served. The created taxi-dispatcher is then simulated in a realistic scenario while avoiding generalization or over-fitting to the area. It is found that an RL-aided taxi dispatcher algorithm can greatly improve the performance of a deployment of SAVs by increasing the overall number of trips completed and passengers served while decreasing the wait time for passengers.

## 1. Introduction

Recent research suggests that the deployment of shared autonomous vehicles (SAV) in the form of fleets of robo-taxis will be a natural and economically feasible way of introducing autonomous vehicles (AV) to the public that will help solve mobility problems of under-served areas and further positively impact network flow [1,2,3,4]. While current deployments of robo-taxis exist, much of the current literature utilizes simple models of AVs, or AV features such as adaptive cruise control (ACC) and cooperative adaptive cruise control (CACC), to extrapolate on the effects of penetration rates for AVs [5]. Furthermore, most reported work in the literature uses grid-based simulations [6] or analytical models [7] to decrease the computational demands of the simulation.

**Figure 1:**
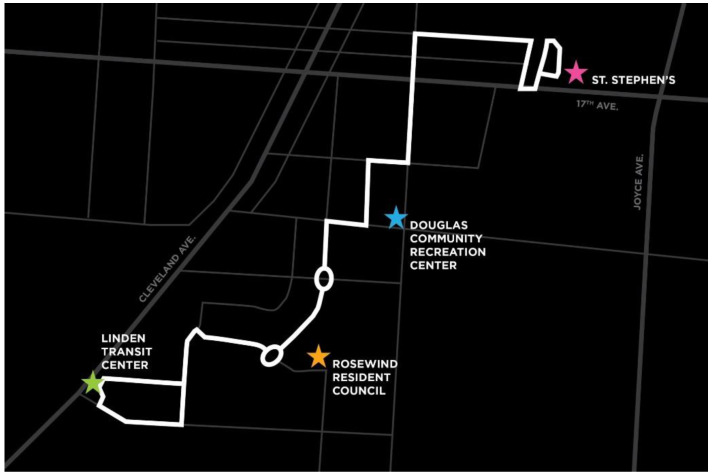
Deployment route for Linden LEAP [15].

Emulating the European project, CityMobil2 [8], the early deployment of low-speed shuttles aimed as last- and first-mile solutions have taken place in Ann Harbor, Michigan [9], Arlington, Texas [10], San Ramon, California [11], and the Metro Phoenix Area [12]. In Columbus, Ohio, the smart circuit deployment of the Smart Columbus project operated in the Scioto Mile downtown area from December 2018 through September 2019 and provided over 16,000 rides with more than 19,000 miles traveled in [13]. The subsequent Linden AV deployment of the Smart Columbus project aimed at closing transportation gaps and providing access to public transportation, affordable housing, healthy food, childcare, recreation, and education was launched in February 2020 and was halted two weeks, due to an on-board incident and continued again afterwards. This project was named Linden LEAP (Linden Empowers All People) and distributed close to 3600 food boxes and more than 15,000 masks during the pandemic [13]. The Linden LEAP deployed autonomous shuttles had a static route and circled between the stops without on-demand capabilities. The static route of deployment is shown in Figure 1. Reference [14] analyzes the challenges faced by the Linden deployment. Some possible issues include slow speed or operation; high delays on route scheduling due to different traffic conditions; the handling of complicated obstacles such as non-signalized intersections; traffic circles and occlusions caused by these; trash pick-up; one street being too narrow for operation; and stopped vehicles and other objects such as trash cans on the road, which may cause stalling of the AV.

Though these issues are common, the deployment of AVs and shared autonomous vehicles (SAVs) is said to reduce vehicle accidents caused by human error. However, new errors may be introduced via actuators or deployment algorithms [16]. Four major areas of benefits with the introduction of AVs have been recognized by the U.S. government: safety, economic and societal benefits, efficiency and convenience, and mobility [17]. Furthermore, the overseeing agency of these laws, the United State Department of Transportation (USDOT), has stated its encouragement of testing and development of AV technologies [18]. However, legislation for the adoption of AV technologies varies drastically from state to state. Reference [19] provides a summary of legislature in the U.S. pertaining to AVs. It is found that, although 41 states considered legislature, only 29 states have passed legislation concerning AVs. It is seen that, although AV technology promises many benefits, the acquisition of such technologies is slow.

Furthermore, from the perspectives of safety and legislation, the introduction of innovative technologies come but without hesitation from the public. Thus, it is imperative for the performance of AVs/SAVs to positively affect the lives of the people using it. This paper models SAVs as an extension of AVs and aims to understand the impact of the deployment these on mobility for the passengers such that their deployment is beneficial. The performance of SAVs with respect to the passengers can be measured by deployment in an under-served or underserved area, passenger wait time for pick-up/drop-off and number of trips completed throughout the day. An optimized SAV dispatcher should increase the number of passengers served while decreasing the wait time. This paper introduces a reinforcement learning approach to achieve these goals.

The main goal and contribution of this paper is the development of a method of modeling and planning of SAV service in a geo-fenced urban area that does not have any public transportation. There are many minor contributions that serve this overall major contribution which are explained next. The developed method presented in this paper first models the traffic network and calibrates it based on available historical traffic data. This is used to generate realistic other traffic in the simulation model of the geo-fenced area where the SAVs will operate. An SAV dispatcher, called the taxi dispatcher here, is then developed to optimize the operation of the SAV service using reinforcement learning to maximize the number of SAV trips and the number of passengers served while minimizing wait times in the presence of the other vehicle traffic which acts as a constraint. This overall approach is both scalable and replicable to other areas of operation and can also be used in a simulation study to determine the optimal size of the SAV fleet and the expected performance of a chosen fleet size based on the metrics mentioned above.

The organization of the rest of the paper is as follows. The relevant literature review is presented in Section 2. This is followed in Section 3 by the modeling of the urban area traffic network and its calibration, to be used for validation, along with the simpler traffic network, to be used for training. Section 4 presents the reinforcement-learning-based optimization method, namely the taxi dispatcher of this paper. The effectiveness of this reinforcement learning taxi dispatcher is shown in Section 5 using the simulation environment developed for validation. The paper ends with conclusions in Section 6.

## 2. Literature Review

The literature on SAVs can be divided into (1) literature regarding studies based on surveys and opinion papers, and (2) studies conducted utilizing simulation for analysis. The former concentrates on public opinion regarding the state of AVs, their adoption, and further possible implications by extrapolating from the information collected through surveys or workshops. Reference [20] provides ample background on the current state of literature regarding SAVs. The literature on adoption of AV technology shows that the way the public will be ready to adopt AV technologies is through the use of shared autonomous vehicles (SAVs). In turn, the factors that will guide the rate of adoption are the level of comfort, time, and cost. Reference [21] is a study composed of a survey of 2588 households with an over-represented demographic of participants between 18 and 24 and under-represented demographic of 65 and above. From their survey, it can be concluded that although young Americans are not confident with the use of AV technologies, opinions about the subject show a promise of rise in shared rides over time. Meanwhile, Reference [22] shows a promise of public acceptance to trade-off their current vehicles with shared autonomous electric vehicles (SAEVs). Likewise, Reference [23] shows partial support for successful adoption of AVs as part of mobility solutions via SAVs. It was also found that the optimization of comfort, cost, and time will increase the demand of SAV use. Reference [24] further agrees on the adoption of the technology favoring SAVs, and it was also found that the important factors to the users for adoption of SAVs are the determination of routes and co-passengers.

Meanwhile, studies regarding simulation of SAVs vary in variables of interest such as vehicle miles traveled (VMT), emissions, price, etc., as well as simulation type and penetration rate. In general, it is understood that the total replacement of vehicles with AVs generates higher road capacities. Furthermore, the performance of SAVs highly relies on the dispatching algorithm. Reference [25] assumed an SAV penetration rate of 3.5% and simulated a gridded city based on Austin. Furthermore, variables such as trip generation rates, and trip relocation strategies were varied. It was found that each SAV can replace 11 conventional vehicles while increasing travel distance on each SAV 10% more than a typical vehicle trip. Reference [26] utilizes a SUMO simulation to evaluate road capacity increase based on a 100% penetration rate of AVs. It was shown in that study that replacement of vehicles with AVs leads to an increase in capacity for existing traffic infrastructures. Furthermore, congestion and loss of time, which consequently increases the quality of the traffic flow, are reduced. However, even a single non-AV is able to create a state of congestion. Reference [27] shows a mixed mobility study with SAV integration. It was found that the mobility demand can be served with 10% of the vehicles. Furthermore, Reference [28] replaces all vehicles in Berlin with AVs and shows that a fleet of 100,000 vehicles is required to serve the demand currently served by 1.1 million vehicles. However, the prices scale higher as the service is given to outskirt locations and remote locations with a single passenger. On a follow-up study, Reference [29] evaluates the potential for shared rides and the introduced algorithm is tested against a real-world data set of taxi requests in Berlin. It is found that vehicle miles traveled (VMT) can be reduced by 15–20%, while travel time increases can be kept at less than three minutes per person. Meanwhile, Reference [30] shows improvement of accessibility with the implementation of shared services as well as the reduction in transport costs. Regarding the performance parameters, Reference [31] shows that there is a strong correlation of performance and fleet size as well as shared or individual use deployment strategy.

On the optimization of the SAV dispatch, the work for optimizing the SAV dispatcher in the literature is limited. Reference [30] simulates an optimized SAV dispatcher by implementing a minimum set cover algorithm. Their algorithm converts the map of Lisbon to a grid simulation and considered all off-street parking facilities less than 5 min away from a station as parking stations for the SAVs. The dispatcher dispatched to multi-modal forms of transport and ran a local search algorithm maximizing the number of passengers’ completed requests of times and modes. Furthermore, the taxi dispatcher tried to insert passengers using the minimum insertion Hamiltonian path. Next, Reference [32] sought to optimize the data collected from vehicle rides in Florida by matching and maximizing number of passengers per vehicle trip while maintaining trip times. In their approach, the authors use on demand dynamic ride sharing in routing algorithms to maximize shared rides. In general, their taxi dispatching approach took the form of allocating as many passengers en route as possible while maintaining time restrictions. Reference [33] utilized the set-up of the interval scheduling problem and the use of the earliest finishing time (EFT) to extend these heuristics to car-sharing. The resulting formulation uses set-up costs, and time windows for jobs and shows that car-sharing allocation is an NP-hard problem. Lastly, Reference [34] used a simple dispatching algorithm that works well enough for simulation purposes, but the authors admittedly showcase room for improvement.

## 3. Modeling

There are many modeling features necessary to implement the realistic performance of a SAV fleet and its dispatcher. These include, the network, the SAV agents, their surrounding traffic, and requests. Additionally, the approach taken in this paper is to train a taxi dispatcher based on reinforcement learning principles. Thus, a second simulation environment must be created where the agent is able to learn and practice dispatching. Thus, first a simulation environment for validation will be created, and then, a simulation environment for training will also be introduced.

### 3.1. Validation Modeling

The Linden network of the Smart Columbus Linden LEAP autonomous shuttle deployment is used here to develop and demonstrate the RL taxi dispatcher. The approach for the validation model of the Linden network is to use an agent-based micro-simulation. Thus, the environment is first built; then, the environment is adapted with a fleet of SAVs, and consequently, the environment is adapted with a taxi dispatcher. The process to build the network is outlined in [35]. OpenStreetMap information was used to create a network version of the Linden area and with further work on intersections and areas of conflict, the network was ready for simulation. Ring and barrier systems were used to control the traffic lights. A ring and barrier system is a way to encode safe pairings of movements and define phases for control of the intersection. It was important to model such behavior as the network used for simulating was prone to traffic jams without the aid of traffic lights.

**Figure 2:**
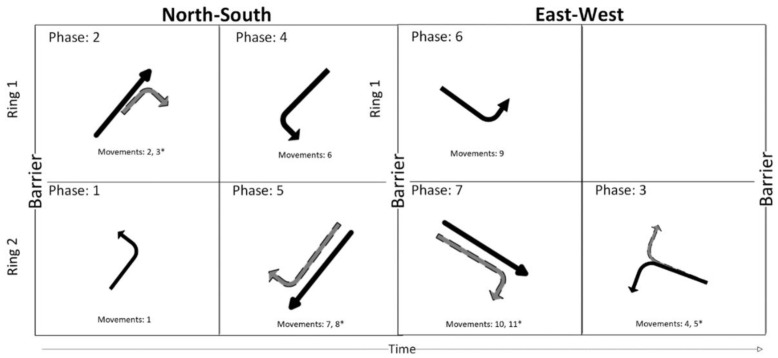
Full Ring and Barrier diagram for Cleveland Avenue and Seventeenth Street.

**Figure 3:**
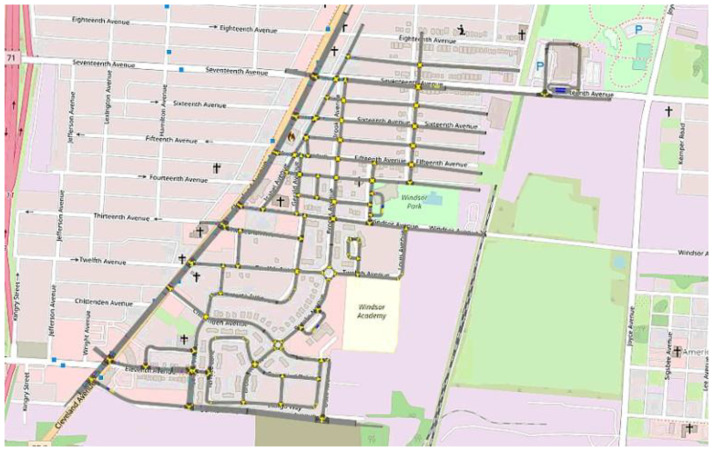
Simulated Area of Deployment.

The first step was to divide the intersection phases into two groups which determine the placement of the first “barriers”. This can be performed by separating the phases into north- south and east–west phases. Next, the phases in each group were sorted based on the compatibility of the phases. That is, phases which are not compatible were further sorted into sequences which are called rings. The beauty of ring and barrier diagrams is that adjusting the timing for the traffic signals becomes easy as the length of the phases is adjusted at will as long as they are maintained inside their corresponding barriers. This allows for more traffic flow by allowing different phases to proceed and to be mix-and-matched. The next task is to decide the length of each phase. For this, the county was contacted for information regarding their traffic lights and these phases were adjusted accordingly. As an example, the diagram for the Cleveland and Eleventh Avenue intersection of the Linden area can be seen in Figure 2. In this way, the entire Linden area was adapted with ring-and-barrier signal intersections based on timings provided by the city on the main road. The map can be seen in Figure 3.

**Figure 4:**
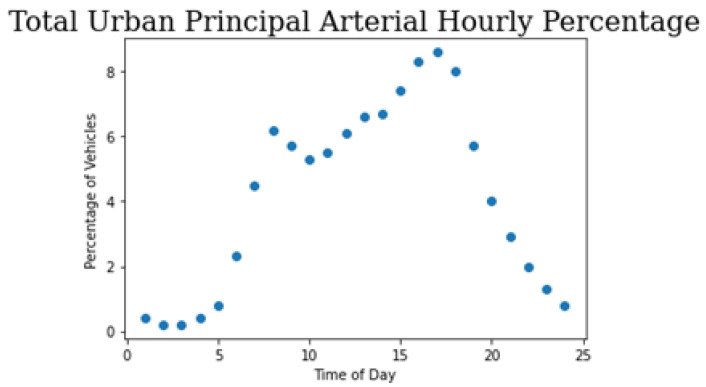
Plot of percentage vehicles vs. time of day.

Furthermore, the network was calibrated based on vehicle capacity, route choice, and system performance. The necessary field measurements were obtained through the Mid-Ohio Regional Planning Commission (MORPC) site [36]. The data, however, were limited so many decisions had to be made to create a tuned network. The method for calibration used was to interpolate the observed information to populate base traffic for the whole network. The necessary vehicle inputs were placed along Cleveland avenue, Bonham Avenue, and Seventeenth Avenue. Next, routing decisions were placed along the network so as to minimize the GEH statistic observed where observed data were provided. The GEH statistic is a goodness-of-fit measure that quantifies the acceptable differences between field observations and simulation observations. An acceptable GEH statistic in a network is <5. Furthermore, temporal dependencies were introduced into the network by interpolating from provided Peak Hour to Design Hour Factor report [37]. This adjusts the total traffic in the network depending on time of day. An example of this distribution for an Urban Principal Arterial road can be observed in Figure 4.

**Figure 5:**
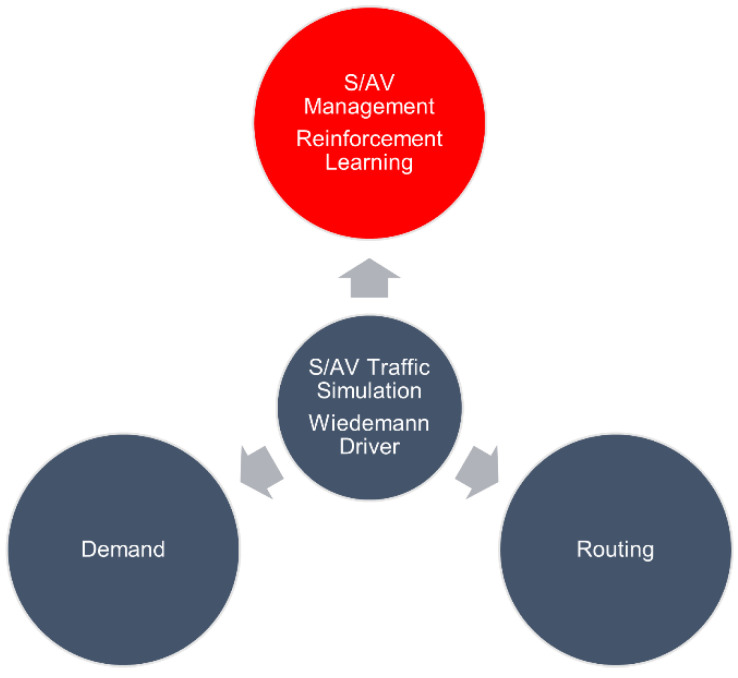
Extension Package Library written in Python [35].

Next, the network was adapted with six SAV pick-up/drop-off locations distributed along the network. The base of the SAVs is assumed to be Stephen’s Community center as this is where the SAVs recharged and stayed during non-working hours during the real deployment. Furthermore, an extension of the simulation was built using the COM interface as is described in [35]. The main working modules include the demand module, which allows the simulation of passengers placing requests; the routing module, which creates the paths for SAV travel; and the SAV management module, which models the dispatchment of SAVs according to different algorithms. These algorithms will be discussed in a later section. The main three modules are interconnected by the traffic and SAV simulation, which updates at the same rate as the rest of the modules. This structure can be seen in Figure 5.

**Figure 6:**
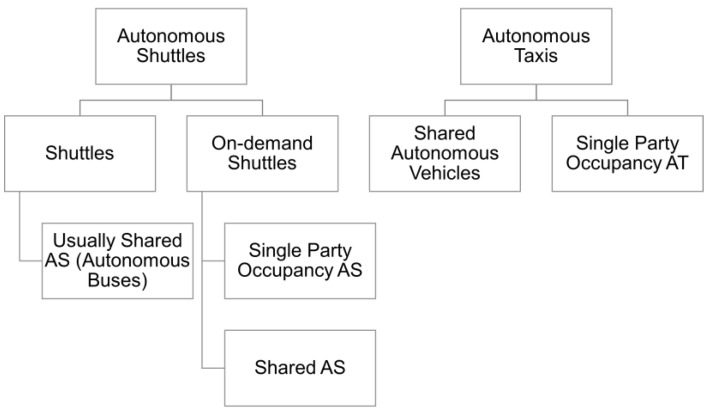
Variations in ATs.

It is important to mention the level of SAVs simulated. In this paper, autonomous taxi (AT) is used often as an umbrella term for any kind of services involving an AV as a means to move passengers between two locations. However, it is recognized that there are many variations of ATs and each one deserves its own detailed model. Hence, in this paper, when referring to autonomous shuttles, or ASs, it is understood that an AS consists of an AV with a fixed set of locations. The order and paths taken to get to and from may vary, but the locations are fixed as it has been indicated in the past by placing “drop-off/pick-up” locations across Linden. Often, the implementations of ASs are on fixed routes due to the limitations of the resulting geo-fencing of the regions. On the other hand, an AT is an AV that may stop at any desired spot through the area. These AVs have to inherently be more advanced than the current level 3 deployments mentioned in the beginning of this paper. Furthermore, an AS may have different kinds of requests. The deployed Linden shuttle was a shuttle operating on a fixed route. By comparison, the types of shuttles modeled in this paper are “on-demand” shuttles, which adapt their on-coming destination based on the passenger demand. A final distinction is that of single party occupancy AS and SAVs. Single party occupancy ASs may serve more than one passenger, but the driving logic will pick up the party and then drop off the party at the selected location while not picking up any new passengers along the way. On the other hand, SAVs will plan their routes according to both picked-up and to-be-picked-up passengers along the way. Both single and shared occupancy AS will be compared in this paper. A summary of these distinctions is show in Figure 6.

**Figure 7:**
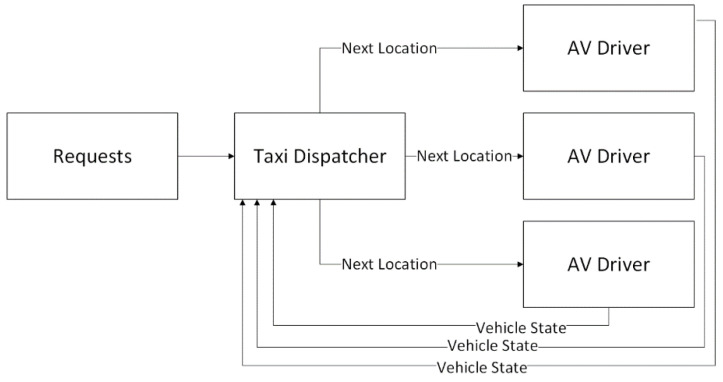
Three SAVs regarded as extensions of AVs by adding a centralized upper-level controller.

The simulation of SAVs begins by modeling an SAV agent. Each agent is regarded as an AV with an upper-level controller. The upper level controller receives requests from passengers with a time-stamp of when they are received. The upper-level controller is regarded as the SAV dispatcher and is tuned with parameters such as maximum wait time for pick-up and maximum wait time for drop-off. At each simulation time-step, requests are placed and the information is updated in the SAV dispatcher. The job of the SAV dispatcher is to send AVs to their destinations such that the number of passengers moved from stop to stop is maximized while the wait times are minimized. In this way, there exists one SAV dispatcher controlling a fleet of AVs and the SAV dispatcher hypothesized is a centralized dispatcher. An example of the regarded SAV dispatcher for three SAVs is shown in Figure 7.

**Figure 8:**
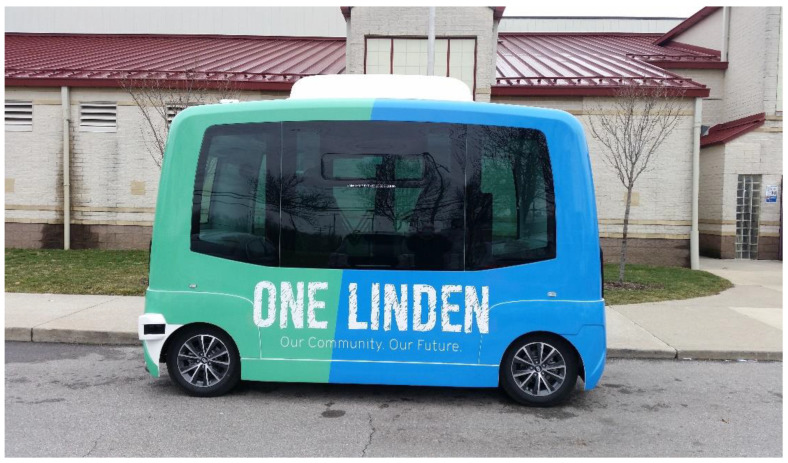
EasyMile Shuttle.

Furthermore, the behavior of interest for the micro-simulation validation model is the parameterized Wiedemann’ 99 model. The Wiedemann’ 99 is a psycho-physical extension model of the stimulus-response model, or the Gazis–Herman–Rothery (GHR) family of models [38]. The GHR models propose that the follower’s acceleration is proportional to the speed of the follower, the speed of difference, and the space headway. The Wiedmann’ 99 model also defines further driver behaviors and parameters to account for the influence of the leading vehicle. Further research has been dedicated to tuning the behavior parameters by analyzing data from deployed AVs. In this paper, the recommended parameters for “Cautious,” “Normal,” and “Aggressive” behaviors outlined in [39] are used. Furthermore, the theorized vehicle model used in this paper is the same model utilized in deployments that were carried out by EasyMile [40]. This model has a passenger capacity per vehicle of 12. In the simulations, the capacity of passengers is extended to 15. A picture of the shuttle in mind is shown in Figure 8.

### 3.2. Training Environment Modeling

**Figure 9:**
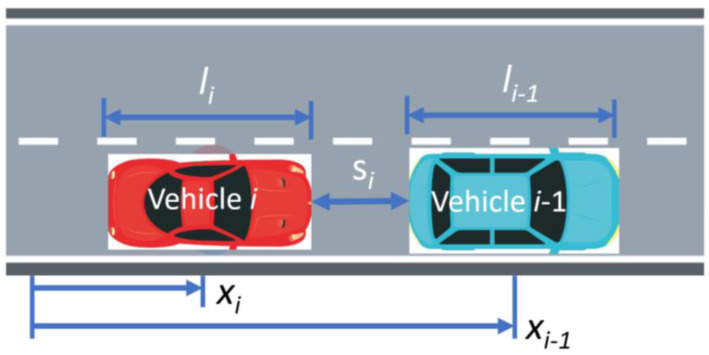
Reference diagram for defined variables.

A second simulation model is introduced which is used for reinforcement training of the dispatch controller. The simulation model for RL is much simpler than the previously introduced environment. Python along with libraries such as Pygame were utilized to build a library in the style of OpenAI Gym [41]. This library was built on top of [42]. The library utilizes a different kind of microscopic vehicle simulator. This model considers the relationship of a lead-following vehicle. The model in question is the Intelligent Driver Model (IDM), which was introduced by [43], and allows for the computation of the acceleration of following vehicle based on the gap between vehicles. As a reference, consider Figure 9, which defines the names of the variables for leader and follower. Table 1 defines the necessary parameters for the IDM model.

**Figure 10:**
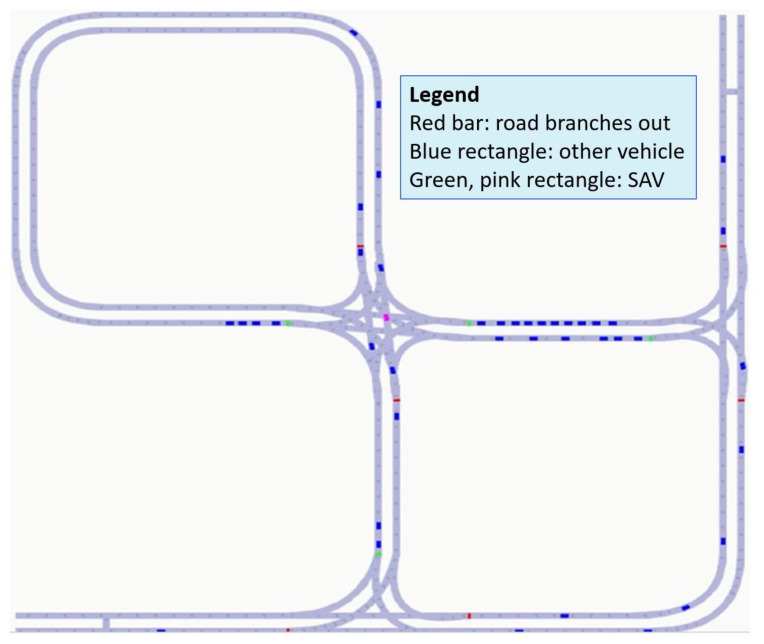
Created RL environment.

The difference between AVs and regular surrounding traffic is that the simulated AV vehicle will behave carefully in relationship to the surrounding traffic. The minimum distance, *s*_0,*i*_, is increased, and the reaction time, *T_i_*, is decreased. Each AV introduced into the simulation was also adapted with a handle such that its behavior can be controlled from outside the simulation. The typical gymAI RL environments provides handles step, reset, and render while declaring the action and observation space. The simulation environment created provides these functions as object handles. Furthermore, pick-up/drop-off spots were also placed along the network as well as traffic light intersections to facilitate the flow of traffic. This resulted in the traffic simulator shown in Figure 10.

## 4. Optimization of Taxi Dispatcher

**Figure 11:**
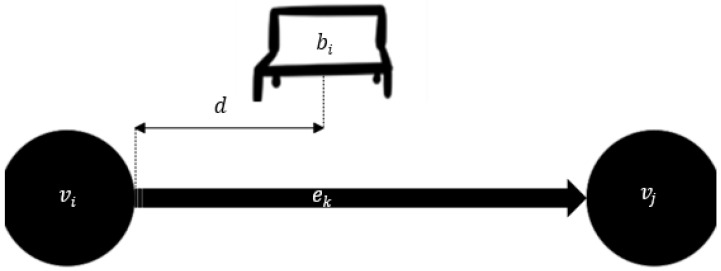
Mathematical representation of SAV pick-up areas placement in network.

The approach to optimizing the SAV dispatcher began by gamifying it. The problem is first set-up as a Markov decision process (MDP). First, assume there is a fleet of *m* SAVs ready for deployment. The network in question can be modeled as a directed connected graph *N* = (*V*, *E*). Throughout the network, setup *n* pick-up/drop-off stops. These are enumerated *B* = {*b*_1_, …, *b_n_*} and can be located by placing it at a distance *d* from the source node, as indicated by Figure 11.

Next, throughout the hours of operation, the SAV travels between pick-up areas *b_n_* attending to the requests of passengers. The state of a taxi, *s*, is then representative of the base in which it is in. Furthermore, to provide the dispatcher with more information, the state *s* is augmented with the state of the requests at pick-up/drop off areas. The pick-up-areas are enumerated *i* ∈ [*m*] and a one-hot encoding representation is utilized where for vector $\vec{\theta }$ each entry $\theta_{i}$ is given by

(1) $$\theta_{i}=\{\begin{array}{l} 1,\;if\;there\;is\;at\;least\;one\;request\;coming\;from\;base\;b_{i}\\ 0,\;otherwise \end{array}$$

Furthermore, let *N_p_* denote the number of passengers in vehicle *i* and *P*_max_ be the maximum number of seats in the vehicle. Then, the state of the vehicle at time *t* is represented by

(2) $$s_{t}=(b_{i},\vec{\theta },N_{p})\;where\;b_{i}\in B,\;N_{p}\in [P_{max}]$$

while updating the simulation, it may be the case that the vehicle is driving. In this case, denote the driving behavior by setting *b_i_* = −1. It will be assumed here that the dispatching process can be modeled as a Markov process so that a Markov decision process can be used later. We are assuming that the next dispatching request attended to depends only on the previous request attended to, which can be expressed mathematically in terms of the given probability state transition *P*

(3) $$P(s_{t}|s_{t}{}_{-1},\ldots ,s_{0})=P(s_{t}|s_{t}{}_{-1})$$

being the same at every point in time or

(4) $$P(s_{t}|s_{t}{}_{-1})=P({s_{t}}^{\prime }|{s_{t}}^{\prime }-1),\;\forall t^{\prime }.$$

by establishing the dispatching process as a Markovian process, where it is easy to see that this leads to a natural transition into a Markovian decision problem. A Markovian decision problem requires an augmentation of a Markovian process with a set of actions and rewards functions. In the case of the dispatch problem, the set of actions is an array containing all the possible routes from the current pick-up spot.

Though not trivial, it is possible to assume that the path chosen from one pick-up spot to another is always the shortest path with respect to distance. This path is found by creating a directed graph representation of the network, where nodes equal intersections and edges equal roads. Then, Dijkstra’s algorithm is applied to find the shortest path. Dijkstra’s algorithm is well-known in the computer science field and creates a tree of paths by calculating the shortest path from one node to every other node [44]. This simple algorithm runs with time complexity *O*(|*E*| + |*V*| log |*V*|), where |*E*| is the number of edges and |*V*| is the number of vertices of the graph representation and suffices to calculate a shortest path. This action is performed at the beginning to plan routes.

Next, the action function is represented by

(5) $$a(s)=s^{\prime };\;s,s^{\prime }\in S$$

where *S* is the set of all possible states. The reward function is represented by

(6) $$r(s,a)=r_{s}{}_{,a};\;r_{s}{}_{,a}\in R$$

The reward function is further crafted as follows. At every step of the Markovian decision problem, the agent decides to take action *a*. This action represents a destination with the knowledge that the SAV is standing at pick-up stop *i* and has requests vector representation $\vec{\theta }$ Furthermore, denote a time limit per request, *T*_max_. This is the maximum amount of time a passenger is willing to wait and is assumed to be fixed. Note that the list of requests is updated at each time step to eliminate requests that have exceeded the maximum wait time. Let *t_req_* be the time the SAV took to serve a request. During a simulation, it is possible that an outdated request pends for some small amount of time if the SAV is deployed before *T*_max_ arrives. Thus, this case is also addressed in the mathematical formulation by considering when *t_req_* ≥ *T*_max_. Then, the request reward function for a single taxi *i*, is

(7) $$r_{i}=\{\begin{array}{cc} -1 & if\;\theta_{i}=0\\ 0 & if\;vehicle\;i\;is\;driving\\ 0 & if\;\theta_{i}=1\;and\;t_{req}\geq \;T_{\max}\\ N_{p}\frac{T_{\max}}{100}\log\left(\frac{T_{\max}}{t_{req}}\right) & if\;\theta_{i}=1\;and\;t_{req}<\;T_{\max}\; \end{array}$$

**Figure 12:**
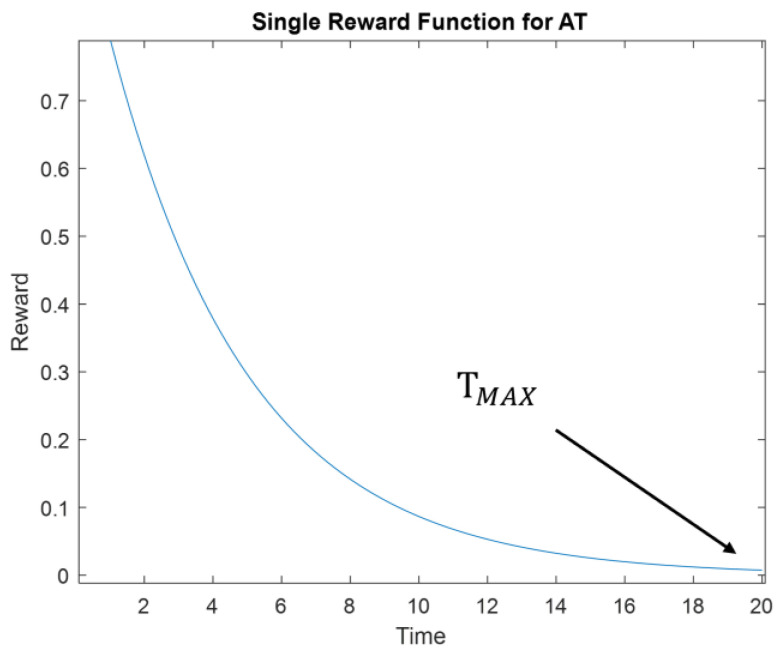
Reward function for taxi dispatcher.

Each reward function only rewards vehicles when they complete the request before the set maximum amount of time. If the vehicle arrives at a destination with no requests, there is a negative reward as this indicates the vehicle has wasted time driving to a place with no requests. While vehicle is driving, it cannot accumulate new rewards. Furthermore, higher rewards go to requests completed earlier and with more passengers picked up. The rewards function for *N_p_* = 1 is shown in Figure 12.

The total reward is the average of the reward collected by every vehicle or,

(8) $$r_{tot}=\sum \frac{r_{i}}{m}$$

Coupling the Markovian decision problem with the reward and action function, the dispatch problem can be represented as an RL problem. The applied formulation is a Q-learning algorithm [45]. Q-learning can be applied to a Markovian decision problem, and it has been shown earlier in this section that a taxi dispatcher agent can be modeled as a Markovian decision problem. Q-learning will then find an optimal policy for the taxi dispatcher according to the specified reward function. Consider a standard Q-learning algorithm with variables reward discount *γ*, learning rate *α* and exploration rate *ε*. The Q-learning update rule is

(9) $$Q(s,a)=(1-\alpha )Q(s,a)+\alpha [r_{tot}+\gamma E_{\pi }(maxQ^{*}(s^{\prime },a^{\prime }))]$$

The corresponding algorithm is shown in Algorithm 1.
**Algorithm 1** Q Learning Algorithm1:Initialize Q-table of size (states, actions)2:Choose reward discount, learning rate and exploration rate3:**Do**4:  Choose random number between 0 and 15:  **If** random number is less than exploration rate6:    Choose random action7:  **Else**
8:    Choose maximum Q-action9:  Perform chosen action10:  Observe11:  Update Q entry based on previously defined Q-update12:**Until**13:  Reward threshold is achieved

**Figure 13:**
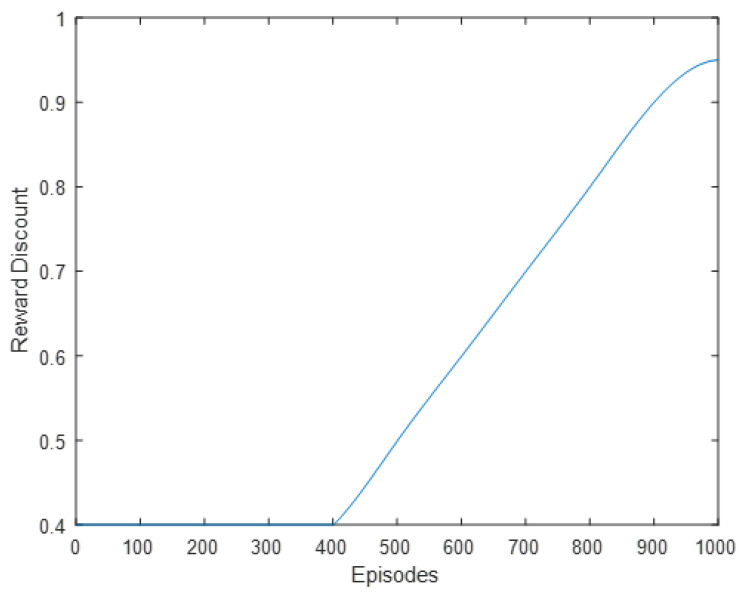
Reward discount plotted over episodes.

**Figure 14:**
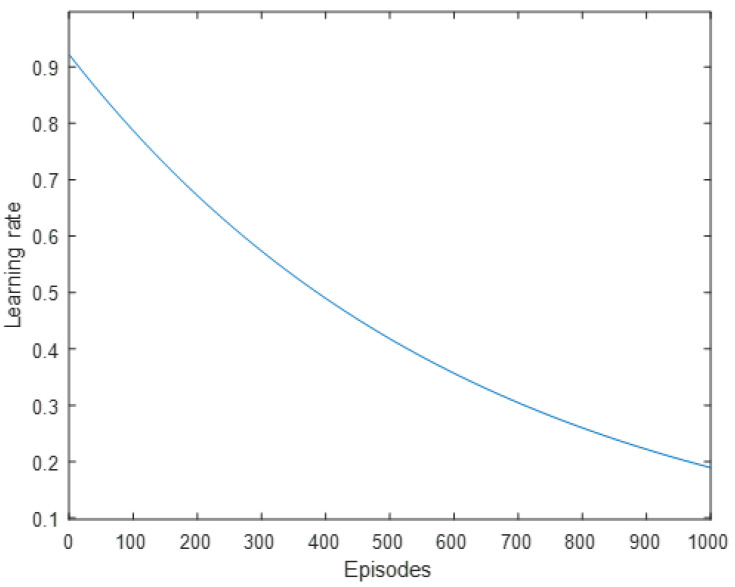
Learning rate plotted over episodes.

**Figure 15:**
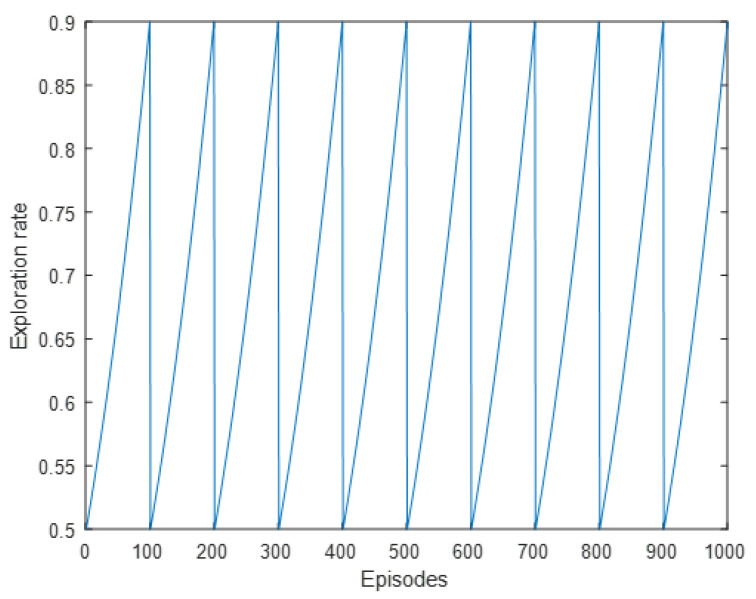
Exploration rate plotted over episodes.

*γ* > 0 is the reward discount and represents how much weight current rewards have vs. past rewards. *γ* is set to 0.4 for the beginning iterations since the algorithm will count the immediate rewards much heavily than long-term rewards. Once training reaches above 400 episodes, it slowly increases *γ* until *γ* = 0.95. This can be observed in Figure 13. *α* > 0 is the learning rate and represents how fast the new Q-value observed is adopted. *α* is slowly decreased over time from 0.9 to 0.2. This can be observed in Figure 14. *ε* > 0 chooses the odds in which a random action is chosen, i.e., the exploration vs. exploitation rate. Figure 15 shows the chosen learning rate over episodes.

## 5. Simulation

**Figure 16:**
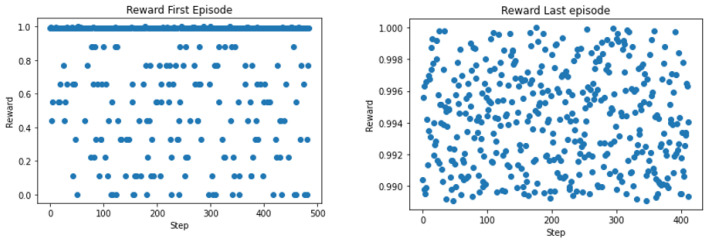
Normalized rewards after training.

The introduced RL algorithm was trained in the Python environment before transfer- ring the learning to the Linden network. The algorithm was trained over 1000 episodes which results in about 500,000 steps. The normalized training rewards for the first and last episodes are shown in Figure 16. The RL algorithm was trained for *m* = 4 and 10 SAV stops. Then, it was extended to encompass the case of six stops, and two- and four-size fleets. The SAV dispatcher can be regarded as a function *f* that maps current requests at time *req_t_* to next deployment destinations for each vehicle *i*, *b_i_*_,*next*_. The variable *req_t_* consists of a three-tuple containing the time the request was placed, *T_reqt_*, the pick-up spot, *b_up_*, and the drop-off spot *b_off_*. Thus, under policy *π*

(10) $$f_{\pi }:(T_{{req}_{t}},b_{up},b_{off})\to (b_{1,next},\ldots ,b_{m}{}_{,next})$$

Policy *π* is dependent on the number of vehicles and shuttle stops. The Linden network was built such that it contains six SAVs. For the case when four shuttles are deployed, it is assumed that for four stops, the state of the one-hot encoding representation is *θ_i_* = 0 at all times. Thus, the SAV dispatcher never sends SAVs to these locations.

When two SAVs are deployed, at each time step, the state of two SAVs is regarded as driving. In other words, for two chosen indices,

(11) $$s_{t}=(-1,\vec{\theta },N_{p}),\;\forall t$$

When six SAV are deployed, two RL dispatchers are used. Initializing dispatchers *f*_1_ and *f*_2_, the overall dispatcher, function *f* under policy *π*, is extended such that each one tends toward requests that will benefit each vehicle’s reward score. Let *f*_1_ control vehicle set *v_f_*_1_ = {*v*_1_,*v*_2_,*v*_3_} and *f*_2_ control vehicle set *v_f_*_2_ = {*v*_4_,*v*_5_,*v*_6_}. This choice was made to make the problem more tractable and to introduce cooperation within vehicle sets. In a multi-agent reinforcement learning approach, each SAV (agent) would be controlled separately, being motivated by its own rewards and taking actions for its own interests as opposed to the interests of the other SAVs.

**Figure 17:**
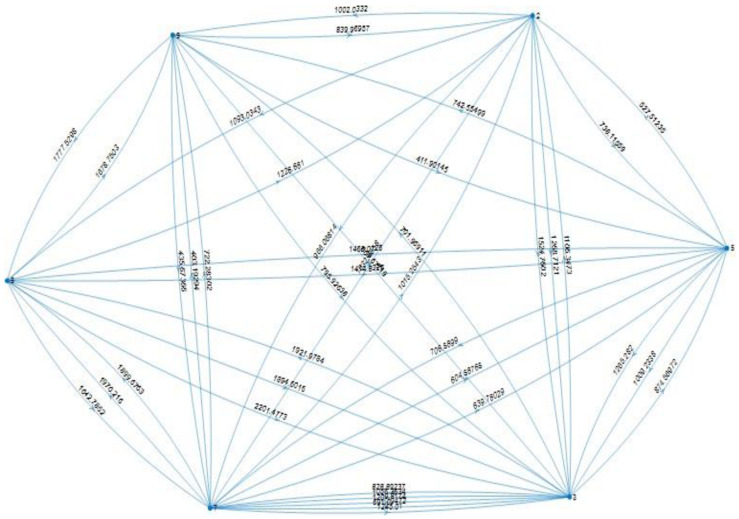
Abstract representation of Linden network.

The handling of the network presented gives the abstract version of the Linden network shown in Figure 17. This is a directed graph with weighted edges equal to the distances between pick-up/drop-off spots in the network. The distance between a vehicle *v_i_* and a request *req_t_*_,*i*_ given its source stop, *b_up_*, *d*(*req_t_*_,*i*_, *v_i_*) is given by the weight of the connected edge from the vehicle’s current pick- up/drop-off spot to *req_t_*_,*i*_. This is always defined as it is computed a priori, at the beginning of the day. Furthermore, it is also assumed that there is always a path between nodes in the graph as shown previously.

The requests *req_t_* are divided into two queries. First, define a radius of *r* = 10 *m*; then, for every request *req_t_*_,*I*_ ∈ *req_t_*, compute set of vehicles *V_r_* such that

(12) $$V_{r}(req_{t}{}_{,i})=\{v_{i}|d(req_{t}{}_{,i},v_{i})\leq r\}$$

Then, create lists *req*^1^ and *req*^2^ such that

(13) $$\begin{array}{l} \exists v_{i}\in (V_{r}(req_{t}{}_{,i})\cup v_{f_{1}})\Rightarrow req_{t}{}_{,i}\in req_{t}^{1}\\ \exists v_{i}\in (V_{r}(req_{t}{}_{,i})\cup v_{f_{2}})\Rightarrow req_{t}{}_{,i}\in req_{t}^{2} \end{array}$$

for enumerated requests *req_t_*_,*i*_ ∈ *req_t_*. For requests *req_t_*_,*i*_ such that *V_r_*(*req_t_*_,*i*_) = ∅, set

(14) $$\begin{array}{l} i\;\;mod2=1\Rightarrow req_{t}{}_{,i}\in req_{t}^{1}\;\\ i\;\;mod2=0\Rightarrow req_{t}{}_{,i}\in req_{t}^{2} \end{array}$$

Based on the defined distribution of requests, an extended version of policy *π* can be described by

(15) $$f_{\pi }=\{\begin{array}{c} f_{1}\;\;\;\;\;if\;req_{t}{}_{,i}\in req_{t}^{1}\\ f_{2}\;\;\;\;\;if\;req_{t}{}_{,i}\in req_{t}^{2} \end{array}$$

**Figure 18:**
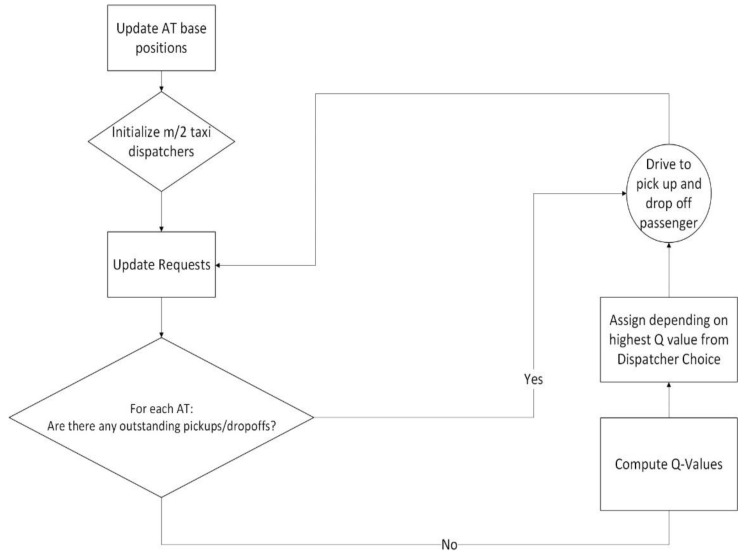
RL SAV dispatcher.

To obtain a deployable algorithm, the list of requests is appended with the drop-offs. Furthermore, a new variable *T_off,MAX_* is defined as a soft drop-off deadline. In the simulations used, half the number of dispatchers as the number of deployed vehicles were used. The completed dispatcher can be seen in Figure 18.

**Figure 19:**
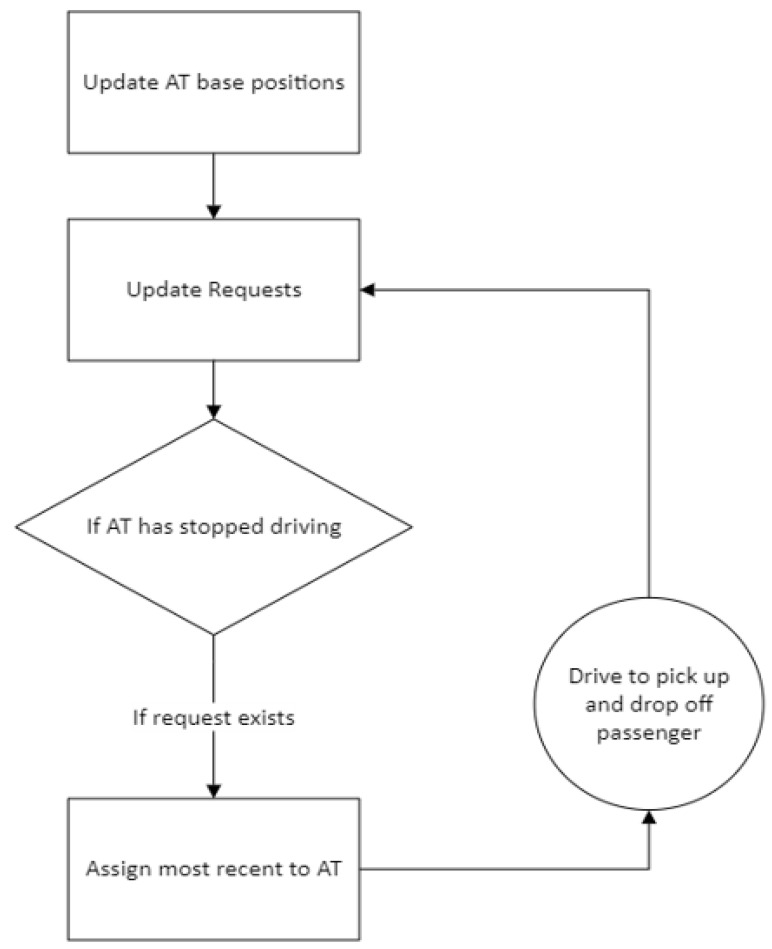
First-come, first serve SAV dispatcher.

The SAV dispatcher is compared to a simple first-come, first-serve algorithm, which provides service to passengers based on the order of requests placed. The decision-making process for serving the passengers can be seen in Figure 19. This simple dispatcher is used for benchmarking purposes. The motivation behind its use for benchmarking in this paper is as follows. The Linden area of Columbus, Ohio, which was modeled in this study, has no public transportation. Two autonomous shuttles operated at very low speeds on a fixed route as a circulator service meaning that they only served one main route shown in Figure 1. This autonomous fixed route circulator autonomous shuttle service, despite its limitation in service area and very long wait and trip times, was found to be very useful in this transportation underserved urban area. Even this simple on-demand first-come, first-serve algorithm that serves the whole geo-fenced area around Linden shown in Figure 3 offers a significant upgrade to mobility as compared to just a fixed route circulator service. It should also be noted that AV technology companies currently offer mobility service that is either a fixed route circulator or on demand first-come, first-serve service on fixed routes. Since our aim is to develop an SAV dispatch algorithm that can be compared with current practice, a first come first serve algorithm was chosen as the benchmark.

The simple dispatcher has no option of shared passengers. Furthermore, the simulations were conducted over a 24 h day period. The first simulation results show the average wait time over all number of trips. Due to the small size of the Linden network, it was found that higher number of AVs deployed could easily cause a traffic jam if the AVs deployed were acting cautiously. This is due to the low-speed of the vehicles. However, this speed was not changed within the “cautiously” deployed AVs since this behavior resembles the currently deployed Linden LEAP shuttles. Thus, it is imperative to pay close attention to the introduced size and behavior of the fleet.

**Figure 20:**
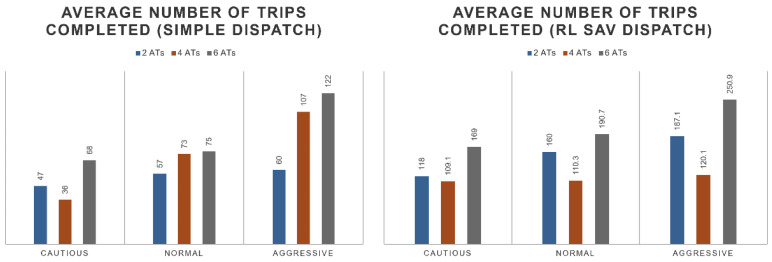
Number of trips of simple dispatcher vs. SAV dispatcher.

**Figure 21:**
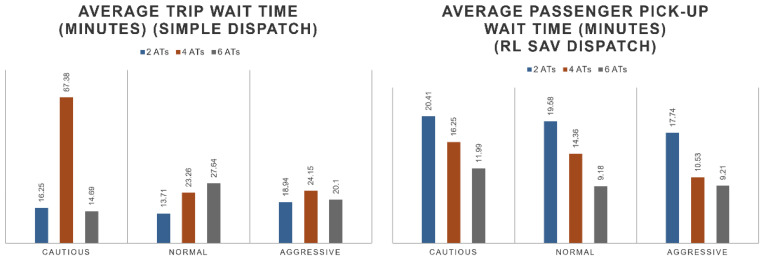
Wait time of simple dispatcher vs. SAV dispatcher.

**Figure 22:**
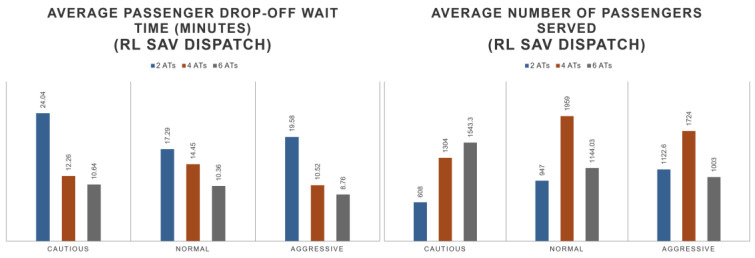
More stats on SAV dispatcher.

For the performance of the dispatcher, it can be observed that the number of completed trips double with the addition of the RL SAV dispatcher in comparison with a simple dispatcher. The dispatcher tends to flatten the performance of the distributed trips. This is shown in Figure 20. The average wait time is also optimized by the RL SAV dispatcher. The average wait time decreases to 10–20 min while the regular dispatcher sees wait times of up to an hour but of an average of 25 min. The fleet wait time performance linearly improves with the size of the fleet. See Figure 21 for details. In Figure 21, the average wait time of 2SAVs of simple dispatch is observed to be less than that of SAV RL dispatch in cautious and normal driving modes. The RL SAV dispatcher tries to maximize number of trips while minimizing wait times. Please note that for the two cautious and normal SAVs, the number of completed trips were much higher as seen in Figure 20 for the RL dispatcher as compared to the simple dispatcher. Since the cautious and normal SAVs have slow speeds, it takes them much longer to serve more trips trying to increase ridership with only two SAVs because of the larger travel distance per SAV that is needed. As the number of SAVs is increased, the higher number helps as the travel distance required per SAV to service more passengers is lower and the wait times are less for the RL dispatcher. It can be observed that the drop-off wait time of the fleet also linearly improves with the size of the fleet. The average number of passengers served is also linear with the size of the fleet. These observations are shown by the numbers in Figure 22.

Using image processing, it is possible to extract useful information about roadways and vehicles travelling on roadways. CAVs and UAVs can benefit from this information in terms of fuel economy, ride comfort, and mobility. Since information regarding traffic flow, average vehicle speed, presence of work zones, as well as queues and accidents can be detected using cameras in conjunction with UAVs, a companion computer setup with a camera was prepared.

## 6. Conclusions, Limitations, and Future Work

Deployment of SAVs can greatly increase the mobility of the people affected by the technology. However, great attention should be paid to details such as size of fleet and expected driver behavior. Furthermore, these technologies should be optimized for deployment based on constraints that affect the passengers. Without well-developed and tested technologies, the deployments of SAVs can harm the perception of new technologies and delay the adoption of such. In this paper, a Q-learning based algorithm was developed, which trained an agent to become a SAV dispatcher. The variability introduced two to six SAVs with improved dispatching logic while also varying the AV driving logic. It was shown that the SAV dispatcher optimization could reduce passenger wait times and increase the number of served taxi requests, thus benefiting the passengers utilizing the service. The number of trips double with optimized logic while wait times also decrease to an average of 15 min. The fleet size is linearly proportional to the wait times, while the average completed number of trips mostly increases by 60 with better driving logic. Furthermore, the number of passengers served improves mostly linearly with the improved dispatching logic. Any gaps and jumps observed within the data can be attributed to the driving logic of the vehicles and the size of the fleet. With a larger size of fleet, the driving logic should side further with an aggressive AV driver as too many too slow vehicles cause traffic jams and slow down times for every vehicle in the network, including other SAVs.

The dispatching logic can further be improved by introducing temporal dependencies. Since the RL agent was trained over variable traffic flow conditions, it is possible to improve the performance by categorizing temporal dependencies and by further creating multiple temporal-dependent behaviors. The independence of training under different network geometries could also introduce further improvements by decreasing the chance of over-fitting. Further limitations include obstacles in the travel links such as traffic jams or closed lanes where the dispatching agent cannot perform decisions. Future work includes extending the dispatching logic to networks where high levels of traffic density affect the vehicle routes or obstacles such as the ones discussed before introduce further choices thereby introducing dynamic route assignments. However, overall an RL-aided taxi dispatcher algorithm can greatly improve the performance of a deployment of SAVs by increasing the overall number of trips completed and passengers served while decreasing the wait time for passengers.

In this paper, the SAVs were modeled at a higher level based on their cautious, normal, or aggressive autonomous driving systems. Our future work will also take a look at these SAVs at a lower level and focus on the controls [46], collision avoidance [47], safety evaluation and energy efficiency [48] of their autonomous driving systems. Robust control methods that have been used for autonomous driving [49,50,51,52] being successful in other demanding applications [53,54,55,56,57] can be used to handle uncertainties in modeling and external disturbances. Safety evaluation will need to use model-in-the-loop, hardware-in-the-loop and vehicle-in-the-loop simulation evaluation.

Reinforcement learning instead of deep reinforcement learning was applied in this paper as deep reinforcement learning was taking an extremely long time to train in the computer used. Our future work will also investigate the use of deep reinforcement learning for solving the optimal SAV taxi dispatcher problem. Some recent examples of deep reinforcement learning applied to the taxi dispatching problem are presented in references [58,59]. It should be noted that these references treat the ride hailing taxi dispatching problem for regular taxis driven by a driver and do not consider autonomous taxis with consideration for their autonomous driving (cautious, normal, and aggressive) nor do they treat the shared autonomous vehicle case with large passenger capacity. The RL SAV dispatcher of this paper solves the problem of increasing the number of passengers served while minimizing wait times for shared rides with passengers picked up from nearby locations while the taxi dispatcher in the abovementioned references does not use ride sharing.

## Figures and Tables

**Table 1:** IDM parameters.

Variable	Definition
*s*_0,*i*_	Minimum distance between vehicle *i* and *i −* 1
*v*_0,*i*_	Maximum velocity of vehicle *i*
*θ*	Acceleration smoothness
*T_i_*	Reaction time of vehicle *i*
*a_i_*	*i*th vehicle’s comfortable acceleration
*b_i_*	*i*th vehicle’s comfortable deceleration
s*	Desired distance between vehicles *i* and *i −* 1

## Data Availability

Not applicable.
